# Multi-Objective Real-Time Tuning of SVC Used in Electrified Traction Systems

**DOI:** 10.3390/s22041584

**Published:** 2022-02-17

**Authors:** Mohammad Hossein Bigharaz, Mehdi Amiri Dehcheshmeh, Hadi Givi, Štěpán Hubálovský

**Affiliations:** 1 Department of Electrical Engineering, Amirkabir University of Technology (Tehran Polytechnic), Tehran 159163-4311, Iran; bigharaz@aut.ac.ir (M.H.B.); dehcheshmeh@aut.ac.ir (M.A.D.); 2 Monenco Iran Consulting Engineers Company, Tehran 1994643315, Iran; 3 Department of Electrical Engineering, Shahreza Campus, University of Isfahan, Iran; h.givi@shr.ui.ac.ir; 4 Department of Applied Cybernetics, Faculty of Science, University of Hradec Králové, 500 03 Hradec Králové, Czech Republic

**Keywords:** current unbalance, multi-objective optimization, reactive power compensation, SVC, traction system

## Abstract

Electric train system is a very large load for the power network. This load consumes a large amount of reactive power. In addition, it causes a massive unbalance to the network, which results in many problems such as voltage drops, high transmission losses, reduction in the transformer output ability, negative sequence current, mal-operation of protective relays, etc. In this paper, a novel real-time optimization approach is presented to adjust the static VAR compensator (SVC) for the traction system to realize two objectives; current unbalance reduction and reactive power compensation. A multi-objective optimization technique entitled non-dominated sorting genetic algorithm (NSGA-II) is used to fulfill the regarded objectives simultaneously. A comprehensive simulator has been designed for electric train network modeling that is able to adjust the parameters of SVC in an optimum manner at any time and under any circumstances. The results illustrate that the provided method can efficiently reduce the unbalancing in current as well as supply the demanded reactive power with acceptable precision.

## 1. Introduction

Nowadays, using railway networks, especially 2 × 25 kV autotransformer type electric traction systems, is increasing in some countries such as Spain, France, Belgium, Italy, Netherland, Russia, etc. [1]. So the power demand for the traction systems is also increasing, which in turn leads to more current unbalance as well as more reactive power consumption [2]. In [3], while the traction vehicle (with three-phase induction motors) is operated, the effects of the operation on power quality in a 110 kV transmission network were studied. Traction systems are generally fed by only two phases of the electrical network. This arrangement causes current unbalance and some problems to the network, such as increasing power losses reducing the efficiency and productivity of the generation, transmission, and distribution of electric energy [4]. Moreover, the current unbalance may lead to system instability [5,6]. To solve the mentioned problems, some solutions have been proposed, such as using Scott [7] and V-V configuration of the traction system transformer for current balancing [8,9], shunt reactors [10], and flexible AC transmission system (FACTS) devices for compensation of the reactive power. Static VAR compensator (SVC) is a widely-used FACTS device in this regard [11]. Reduction in the co-phase traction power supply (CTPSS) capacity and improving the power quality of freight railways can be realized through suppression of negative-sequence current and compensation of reactive power. To achieve these goals, voltage imbalance compensation was carried out in [12] for the stator of doubly-fed induction generators (DFIGs) utilized in power networks with high penetration of wind generators.

Many investigations in the literature have focused on SVC implementation for the traction system applications. In [5], a combination of thyristor-switched shunt reactor and current source converter was used for reactive power compensation in the traction system. In [13], SVC was used for current balancing through the elimination of negative sequence current. The effects of SVC implementation on the power quality improvement in the Shanghai electric railway network were analyzed in [14] using PSCAD software. In [15], a new method was proposed to predict the load current unbalance with negative sequence current in the AC traction network. Power quality problem in the DC traction system was analyzed in [16], and by using a modified SVC, the network distortion was reduced, which led to voltage profile improvement. Performance of the SVC in the traction substation of the Beijing–Shanghai electrified railway was studied in [17]. Characteristics of harmonic and negative sequence currents were analyzed in [18] to provide the optimum arrangement of SVC installation for low-voltage side of traction substation. Combination of SVC and hybrid power quality conditioner (HPQC) was proposed in [19] to improve the power quality compensation capability in AC electrified railway systems. In this technique, the reactive power demand of the load is met by SVC, while HPQC helps to supply the remaining required reactive power to mitigate the current unbalance problem. In [20], a new electromagnetic hybrid system and a magnetic static VAR compensator (MSVC) were provided to compensate for the power quality parameters of the network. In [21], the authors adopted an integrated compensation technique to mitigate power quality problems encountered in railway power distribution networks. In this approach, DQ decoupling technique was utilized to achieve a double closed-loop control strategy, by which harmonic current detection, as well as reactive power compensation, can be accomplished. In [22], the operation of a half-bridge railway power conditioner (HB-RPC) was analyzed for decreasing power quality problems including reactive power, harmonics, and negative sequence current in railway system. Moreover, a Genetic Algorithm (GA)-based control approach was adopted to achieve an optimized performance. In [23], a novel type of power quality compensation system with two sets of thyristor-controlled reactors and two sets of a thyristor-controlled third filter was presented. The authors claimed that the proposed power quality compensator has some features such as small capacity, low cost, reduced capacity of the active part, and favorable performance for compensating the negative sequence current. To overcome voltage dip effectively, a static VAR compensator was proposed in [24], which was paralleled with a 50 km section of a traction transmission line supplied by local substation. A nonuniform power factor partial compensation approach was presented in [25] to reduce the current rating of railway power conditioner (RPC). This purpose was realized by using particle swarm optimization (PSO) algorithm to specify lower boundary of the grid side power factor. In most of the mentioned works, the authors focused on only one objective for compensation and neglected other advantages.

The monitoring power supply of the electric traction system is an important concern for transportation infrastructures. In this regard, a monitoring system has been proposed in [26], which is capable of detecting short circuit fault as well as electro-erosion of the circuit breakers. This system acquires electrical parameters of the railway through a hardware-software structure combined with a global system for mobile communications (GSM).

In this paper, an SVC is used in the traction substation in order to improve two independent objectives simultaneously. The first objective is reducing the network current unbalance and the second objective is considered reactive power compensation. A multi-objective evolutionary optimization approach entitled NSGA-II is utilized for this purpose. After defining the desirable Pareto front, a Lagrange equation is utilized for selecting the final individual. As a case study, a simulator of the 2 × 25 kV autotransformer type electric traction system, including some moving trains with various operational modes (motoring, coasting, and braking), is used.

The rest of this paper is organized as follows. Section 2 presents a concise overview of the simple 2 × 25 kV AC traction system. In Section 3, the general configuration of the SVC is explained. Section 4 is assigned to the proposed multi-objective optimization. In Section 5, the simulation results of this investigation are presented. At the end, the main conclusions are given in Section 6.

## 2. Arrangement of AC Traction System (2 × 25 kV)

**Figure 1:**
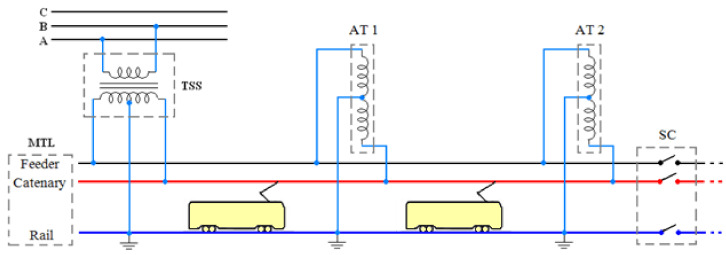
General configuration of the traction system.

The general arrangement of 2 × 25 kV AC traction system is shown in Figure 1. This system includes a multi-transmission line (MTL), autotransformer (AT), traction substation (TSS), trains, and sectioning cabin (SC). The traction system is powered by two phases of the electrical network (e.g., 230 kV) and with a center-tapped transformer, which energizes the electric railway conductors means Catenary (+25 kV), Rail (0 V), and Feeder (−25 kV). The AT is installed at every 8–15 km of the MTL. This method of supply imposes a big unbalance on the network, and due to the moving trains, the system demands variable reactive power in each time period [27,28,29]. Every part of the electric railway system is described as follows.

### 2.1. Train Modeling

In this paper, the train is considered as a current source, and its value would be updated at every moment based on some parameters according to the following equation:

(1) $$I_{Tr}=mode\cdot \frac{f\cdot v}{\eta \cdot e}\left[1+j\tan(\phi \right)]$$

(2) $$\eta =\frac{P_{mec}}{P_{elec}}$$

Newton’s second law can be applied to the train’s movement:

(3) $$M_{eff}\frac{d^{2}x}{dt^{2}}=f-R-G\left(x,v\right)$$

The parameters in the above equations are described as:
modeControl mode of train;fTractive effort (N·m);vSpeed of train (m/s);ηFactor of efficiency;eVoltage of catenary (V);ϕCurrent and voltage phase difference;$P_{mec}$Mechanical power (Watt);$P_{elec}$Electrical power (Watt);$M_{eff}$Train’s mass (effective) (kg);RResistance of train (N);gGravity force (N);θtrack slope;xTrain’s position (m);GTrack resistance (N).

If e=0, the train is in a coasting state, and if 0<mode≤1, the train is in powering and cruising states, and if −1≤mode<0, the train is in the regenerative braking state. The maximum acceleration of a train can be defined by the following formula:

(4) $$a_{max}=gPm.\mu$$

where *Pm* is the ratio of motoring axles, and μ is the frictional coefficient. In railway’s rolling stocks, μ is based on the rail frictional condition and is generally limited to (0.05, 0.5). It may be bigger than 0.5 while the rail is dry or sandy.

For train resistance, some investigations have proposed the empirical relationship (5):

(5) $$R=A+Bv+Cv^{2}$$

where *A* (kN), *B* (kNs/m), and *C* (kNs^2^/m^2^) are known as Davis coefficients. These empirical coefficients are determined by the train’s manufacturer.

In this paper, the following state equations are considered for train’s movement under all possible conditions throughout the track:

(6) $$\left[\begin{array}{c} \overset{\cdot }{x_{j}}\\ {\overset{\cdot }{v}}_{j} \end{array}\right]=\left[\begin{array}{c} v_{j}\left(t\right)\\ \frac{1}{M_{eff}}\left(f\left(mode\left(t\right),v\left(t\right)\right)-R-G\right) \end{array}\right]$$

(7) $$mode\left(t\right)\in \left[-1\;,\;\ldots ,\;1\right]$$

### 2.2. Multi-Transmission Line (MTL) Modeling

**Figure 2:**
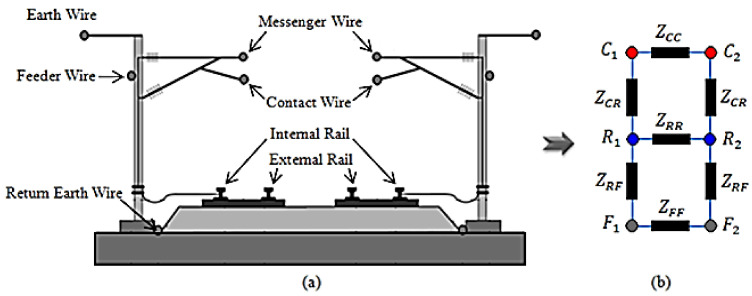
A typical view of MTL system (double lines): (**a**) conductors’ arrangement; (**b**) simplified impedance model.

According to Figure 2, because of the existing many conductors, electrical modeling of the MTL will be difficult. Although various methods have been presented for MTL modeling, a reduction method provided by A. Mariscotti [30] is used in this paper. In this method, three main categories are determined based on the voltage and all conductors are divided into these categories.

### 2.3. Modeling of Autotransformer (AT) Substations

**Figure 3:**
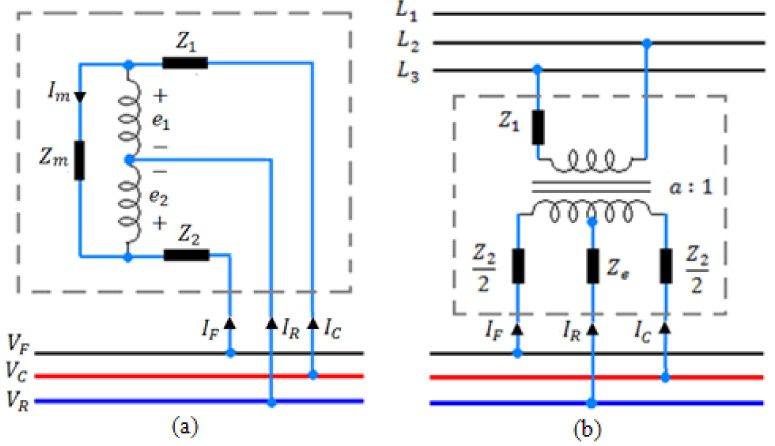
(**a**) The impedance model of AT and (**b**) the impedance model of TSS.

This is a special transformer with equal winding turns ratios (a = 1) and coupled windings with three terminals, which are connected to the conductors of catenary, rail, and feeder throughout the line. Using ATs brings many advantages to the electric railway system, such as: reducing the number of required traction substations along the line, minimizing the electromagnetic interfaces (EMIs) between power electronic equipment and nearby telecommunication circuits, reducing voltage drop of the line, and decreasing the current that may be strayed to the underground’s metal constructions of the rail lines, and eventually reducing the power loss. In this paper, an impedance model of AT is used. As seen in Figure 3a, by using Kirchhoff laws, the following impedance equation for AT can be determined.

(8) $$\left[\begin{array}{c} V_{C}\\ V_{R}\\ V_{F} \end{array}\right]=\left[\begin{array}{ccc} Z_{a} & Z_{a} & Z_{a}\\ Z_{a} & -Z_{m} & 0\\ Z_{a} & 0 & -Z_{a} \end{array}\right]\left[\begin{array}{c} I_{C}\\ I_{R}\\ I_{F} \end{array}\right]$$

(9) $$Z_{a}=-Z_{1}-2Z_{m}$$

In these equations, *Z*_1_ and *Z*_2_ are the leakage impedances of the windings ($a=1\to Z_{1}=Z_{2}$) and *Zm* is the magnetizing impedance.

### 2.4. Modeling of Traction Substations (TSS)

According to Figure 3b, traction substations energize the electric railway system. The transformer of this substation is a center-tab two-phase transformer, for which the primary side is connected to two phases of the electric network, and the secondary side with a 180-degree phase difference is directly connected to the electric railway system. The impedance equation of the TSS can be determined as follows:

(10) $$\left[\begin{array}{c} I_{C}\\ I_{R}\\ I_{F} \end{array}\right]=I_{SS}\left[\begin{array}{c} 1\\ 0\\ -1 \end{array}\right]+{\left(Z_{SS}\right)}^{-1}.\left[\begin{array}{c} V_{C}\\ V_{R}\\ V_{F} \end{array}\right]$$

(11) $$Z_{SS}={\left[\begin{array}{ccc} y_{a}+y_{b} & 2y_{b} & y_{a}-y_{b}\\ 2y_{b} & -4y_{b} & 2y_{b}\\ y_{a}-y_{b} & 2y_{b} & y_{a}+y_{b} \end{array}\right]}^{-1}$$

(12) $$y_{a}=\frac{1}{Z_{1T}+Z_{2}}\overset{}{},\underset{}{}y_{b}=\frac{1}{Z_{2}+4Z_{e}}$$

(13) $$I_{ss}=\left(\frac{V_{PT}}{2a}\right)$$

(14) $$Z_{1T}=\frac{Z_{1}}{{\left(2a\right)}^{2}}$$

*V_PT_* represents the voltage of the primary side, *a* is the transformer ratio, *Z*_1*T*_ is the leakage impedance of the primary side, *Z*_2_ is the leakage impedance of the secondary side, and *Z_e_* denotes the grounding impedance.

**Figure 4:**
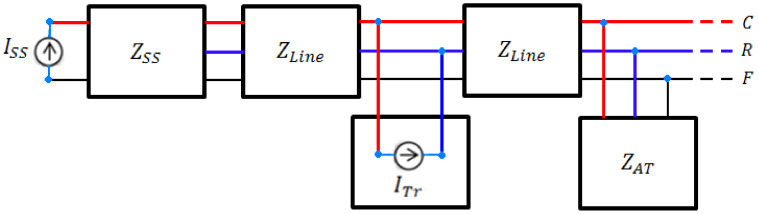
Block diagram model of the electric railway system.

**Figure A1:**
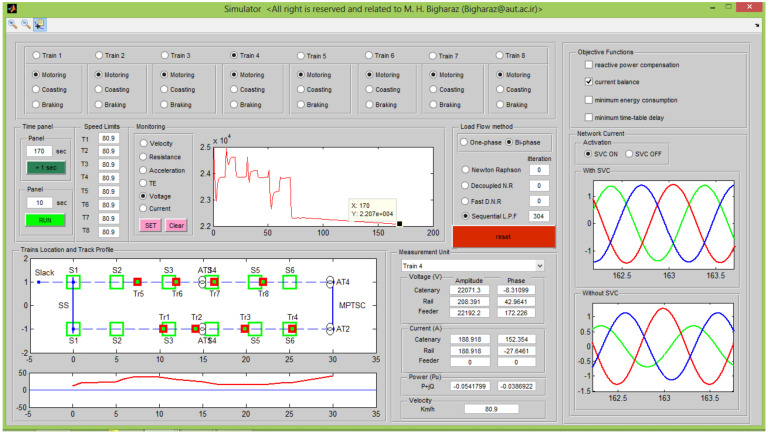
Simulator window specifications developed for electric traction system [28].

After defining the impedance models of all parts of the systems, means trains, autotransformer substations, traction substations, and multi-transmission lines, by composing these models as seen in Figure 4, a dynamic impedance model of the electric railway system can be determined. This dynamic impedance model is a central core for electric load flow calculation of the simulator developed for electric railway system analysis. The developed simulator is briefly described in Appendix A and in Figure A1.

## 3. SVC Implementation

SVC could be considered as the most popular FACTS device with two specific properties: (a) despite all its constructed elements being passive, it can distribute active power between three phases correctly, (b) although SVC is made of the elements with constant impedance, it is able to establish variable impedance in the network [31,32]. Specifically, in the traction system applications, SVC is required in order to fulfill these purposes (a) load current balancing and power factor correction, (b) adjusting the system positive sequence current in an acceptable range, (c) controlling the negative sequence current magnitude in a selectable range [33,34,35].

From an economic point of view, traditionally, in order to improve the unbalancing in the current and also to compensate reactive power in a traction system, two separate pieces of equipment are needed. For example, for improving the current unbalancing, a specific device like Scott, V-V, or open delta transformer is utilized and for reactive power compensation, using a FACTS device is inevitable. Equipping a traction system with both devices is costly. In the current research, both aims, including reducing unbalancing in the current and reactive power compensation are realized with only an advanced SVC that has remarkably lower price than two separate equipment. It should be noted that, among FACTS devices, SVC has lower price compared with other complex FACTS devices in combined series-shunt controllers category.

### 3.1. Network Current Balancing

**Figure 5:**
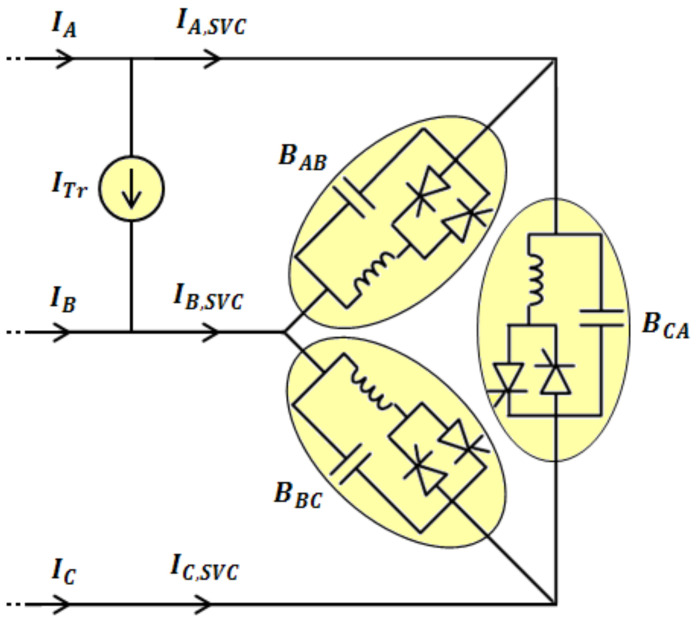
SVC and single train view.

As mentioned already, the traction system has some trains with various operational modes. For the sake of simplicity, equations of the currents are determined in this part only for one train, but in the simulations, some trains with various conditions will be considered in the comprehensive simulator. All electrical parameters for SVC adjustment are determined by using an advanced load flow analyzer. According to Figure 5, the train has been located between phases A and B, then the SVC and the network currents are calculated as follows [36,37]:

(15) $$\left[\begin{array}{c} I_{A,SVC}\\ I_{B,SVC}\\ I_{C,SVC} \end{array}\right]=j\sqrt{3}V_{1A}\left[\begin{array}{ccc} \xi & 0 & \xi^{-1}\\ \xi^{-5} & \xi^{-3} & 0\\ 0 & \xi^{3} & \xi^{5} \end{array}\right]\left[\begin{array}{c} B_{AB}\\ B_{BC}\\ B_{CA} \end{array}\right]$$

(16) $$\left[\begin{array}{c} I_{A}\\ I_{B}\\ I_{C} \end{array}\right]=j\sqrt{3}V_{1A}\left[\begin{array}{ccc} \xi & 0 & \xi^{-1}\\ \xi^{-5} & \xi^{-3} & 0\\ 0 & \xi^{3} & \xi^{5} \end{array}\right]\left[\begin{array}{c} B_{AB}\\ B_{BC}\\ B_{CA} \end{array}\right]+\left[\begin{array}{c} I_{Tr}\\ -I_{Tr}\\ 0 \end{array}\right]$$

where *I_A,SVC_*, *I_B,SVC_*, and *I_C,SVC_* denote the 3-phase line currents of the SVC. *B_AB_*, *B_BC_*, and *B_CA_* are the variable susceptances of the delta-connected SVC, *V*_1*A*_ is the positive sequence voltage of phase *A*, which is considered as the reference voltage, and *ξ* is equal to 1<30. *I_A_*, *I_B_*, and *I_C_* represent the 3-phase network currents, and *I_Tr_* indicates the train current. By decomposing the three-phase currents to positive, negative, and zero sequences, it can be shown that the load current includes only positive and negative sequence currents. The negative sequence current is a significant factor in the network current unbalance. Though, in order to balance the load current, it is necessary to limit the negative sequence current in a defined range. This procedure is carried out through the regulation of SVC susceptances for any condition of the system.

Assuming the train current to be $I_{Tr}=\left|I_{Tr}\right|<\varphi$ and a=1<120, the following equations are used to obtain the triple sequences currents:

(17) $$\left[\begin{array}{c} I_{1}\\ I_{2}\\ I_{0} \end{array}\right]=\frac{1}{3}\left[\begin{array}{ccc} 1 & a & a^{2}\\ 1 & a^{2} & a\\ 1 & 1 & 1 \end{array}\right]\left[\begin{array}{c} I_{A}\\ I_{B}\\ I_{C} \end{array}\right]$$

where *I*_1_, *I*_2_, and *I*_0_ are the positive, negative, and zero sequence currents, respectively. Subsequently:

(18) $$\left[\begin{array}{c} I_{1}\\ I_{2}\\ I_{0} \end{array}\right]=V_{1A}\left[\begin{array}{ccc} \xi^{3} & \xi^{3} & \xi^{3}\\ \xi^{5} & \xi^{-3} & \xi \\ 0 & 0 & 0 \end{array}\right]\left[\begin{array}{c} B_{AB}\\ B_{BC}\\ B_{CA} \end{array}\right]+\frac{1}{\sqrt{3}}\left|I_{Tr}\right|\left[\begin{array}{c} 1∠\phi -30\\ 1∠\phi +30\\ 0 \end{array}\right]$$

By separating *I*_1_ and *I*_2_ into their real and imaginary components, the following equation is derived:

(19) Re(I1)Im(I1)Re(I2)Im(I2)=V1A000001110−32032012−1120BABBBCBCA+13ITrcos(ϕ−30)sin(ϕ−30)cos(ϕ+30)sin(ϕ+30)

Consequently, the negative sequence current (*I*_2_) can be controlled in a specified range. The above equation is used to obtain the SVC succeptances. The equivalent succeptances are a function of the trigger angles and can be expressed as [38]:

(20) $$\frac{\delta_{i}{}^{3}}{3!}-\frac{\delta_{i}{}^{5}}{5!}+\frac{\delta_{i}{}^{7}}{7!}-\cdot \cdot \cdot =B_{i}\pi \omega t$$

where $i\;\epsilon \;\left\{AB,\;BC,\;CA\right\}$ and δ denotes the trigger angle of the thyristor.

### 3.2. Reactive Power Compensation

**Figure 6:**
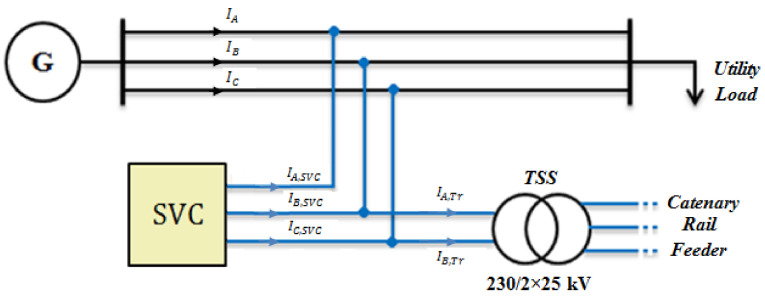
SVC location in the network.

The reactive power needed for supplying the traction system changes alternatively. This is due to the variable nature of the load current based on the trains’ operating condition. For example, when many trains operate in the accelerating mode simultaneously, more reactive power is demanded from the network. This reactive power causes some difficulties such as power losses, limiting the network capacity, etc. Therefore, it is desirable to supply the required reactive power locally through implementing an SVC, as shown in Figure 6.

## 4. Multi-Objective Optimization

Generally, a multi-objective optimization problem (MOP) is considered as a set of ‘P’ objective functions *f= (f*_1_*,…,f_P_)* and a set of ‘*U*’ possible solutions in the decision space. Assume that *Z = f (U)* is an objective space in which every objective must be minimized. For every solution that means *u∈* *U*, an objective string *z∈* *Z* has been allocated. *Z = {z*_1_*,…,z_P_ }* is calculated based on the function *f: U→Z* such that *Z = f (U) = (f*_1_ *(u),…,f_P_ (u))*. The objective string *z* *ϵ* *Z* dominates *z’∈* *Z* if *z_i_ ≤ z_i_’*, *∀ i* *∈* *{*1*,…, P}* and also *∃ j∈* *{*1*,…,P}, z_j_ < z_j_’*. The decision string *u* *∈* *U* dominates *u’∈* *U* if *f (u)* dominates *f (u’)*. On the other hand, a solution *u* *∈* *U* is named an efficient or Pareto optimal member if it is mapping on the objective space is a non-dominated value. The main goal is to identify a proper set ‘*U*’ of efficient solutions that satisfies a minimum deviation with the ideal Pareto front. Recently, evolutionary algorithms have been widely utilized for solving such problems [27].

### 4.1. Problem Definition

As mentioned in the previous parts, two objectives are tracked in this paper, network current balancing (*f*_1_) and reactive power compensation (*f*_2_), as expressed in (21) and (22). In this regard, some constraints are taken into account according to (23) and (24).

(21) $$f_{1}=\sqrt{\begin{array}{c} {\left(I_{A}+a^{2}I_{B}+aI_{C}\right)}^{2}+{\left(1-\left|I_{A}\right|\right)}^{2}\\ +{\left(1-\left|I_{B}\right|\right)}^{2}+{\left(1-\left|I_{C}\right|\right)}^{2} \end{array}}$$

(22) $$f_{2}=\|Q_{demand}-Q_{SVC}\|$$

(23) $$-\lambda <I_{2}<\lambda$$

(24) $$B_{min}<B<B_{max}$$

where *Q_demand_* denotes the reactive power demanded by the traction system, *Q_SVC_* is the reactive power generated by the SVC, *I*_2_ represents the negative sequence current, *λ* is the marginal amount of *I*_2_, and *B* is the SVC’s susceptance, which is restricted between *B_min_* and *B_max_*. These limits are defined according to the SVC’s construction.

### 4.2. Non-Dominated Sorting Genetic Algorithm

Non-dominated sorting genetic algorithm (NSGA-II) refers to a multi-objective optimization strategy that was introduced first by K. Deb in 2002 [39]. This algorithm is similar to the common genetic algorithm (GA) but with two additional functions entitled non-dominated sorting and crowding distance. First, the non-dominated individuals by others are located in the first group, then this group is deleted, and the above process is repeated for other individuals until all of them are rated. Crowding distance is a control parameter, which is calculated for each member of each group. This parameter indicates a degree of closeness to the other members in a group, as expressed in (25).

(25) $$d_{j}\left(k\right)=\sum_{i=1}^{p}\frac{f_{i}(k-1)-f_{i}(k+1)}{f_{i}^{\max}-f_{i}^{\min}}$$

In (25), *k* is the member number existing in the *j_th_* group, and *d_j_(k)* is the crowding distance of this member. *f_i_* represents the *i_th_* objective function for a problem with P objective functions. Cross-over and Mutation are done as classical genetic algorithms.

Two significant properties of NSGA-II that lead to the selection of this algorithm in this investigation are running speed and also a great diversity of solutions along the Pareto front. The running speed of this algorithm is too high in comparison to the other multi-objective algorithms, and this property is obtained by the elitism performance of this algorithm. In this study, due to using the optimization strategy in a real-time manner, the running speed of the optimization algorithm is of great importance. Moreover, the selection strategy of the algorithm, which is non-dominated sorting of population, leads to a fine diversity of solutions on the Pareto front.

**Figure 7:**
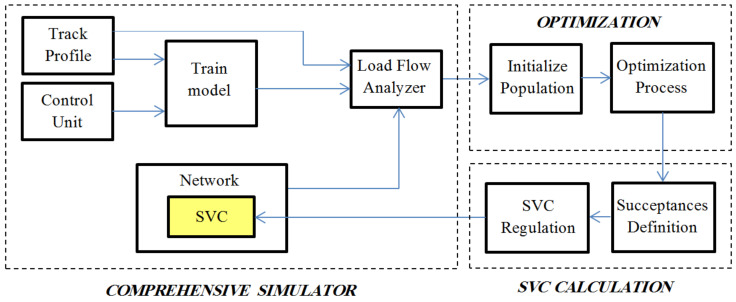
Block diagram of the proposed system.

In NSGA-II, the individuals selected for the next iteration are based on the non-dominated sorting and the crowding distance parameters. More information in this regard can be found in [40,41]. The Block diagram model of the proposed system for regulation of SVC is shown schematically in Figure 7.

In real use of the proposed system, the initial data required for the system will be provided by some sensors, including voltage, current, and phase sequence sensors. These sensors will be located on the main busbars of traction substations and sense and provide the required data at every predefined moment for the optimization block. Finally, by determining the susceptance and regulation, the SVC will be tuned.

## 5. Simulation Results

Specifications of the traction substation (TSS), autotransformer (AT), and the trains in the simulation are presented in Table 1. In order to illustrate the current unbalance properly and to consider traction systems with considerable impact on the network, it is assumed that the traction system consumes 10 percent of the network power. This helps to validate the capability of the proposed SVC regulation strategy. In this paper, the priority of the SVC operation is assigned to the current balancing before power compensation. Therefore, in each time period (1 sec), the best solution is selected from the Pareto front using (26).

(26) $$\min(\lambda_{1}.Obj_{1}\left(i\right)+\lambda_{2}.Obj_{2}\left(i\right))\underset{}{},\underset{}{}i=1,\cdot \cdot \cdot ,npop$$

where *Obj_1_* indicates the first objective (*f*_1_ according to (21)) and *Obj_2_* represents the second objective (*f*_2_ according to (22)). The coefficients *λ*_1_ and *λ*_2_ are weighting factors, which are applied in order to allocate preference between the objectives. Here *λ*_1_ is the weighting factor of current balancing and *λ*_2_ is the weighting factor of reactive power compensation, which are considered to be 0.9 and 0.1, respectively. It should be noted that *λ*_1_ and *λ*_2_ are generally determined by the managing parties of railway and electric network organizations. These factors may be changed during the day based on the parties’ decisions. In (26), npop represents the number of population members.

**Figure 8:**
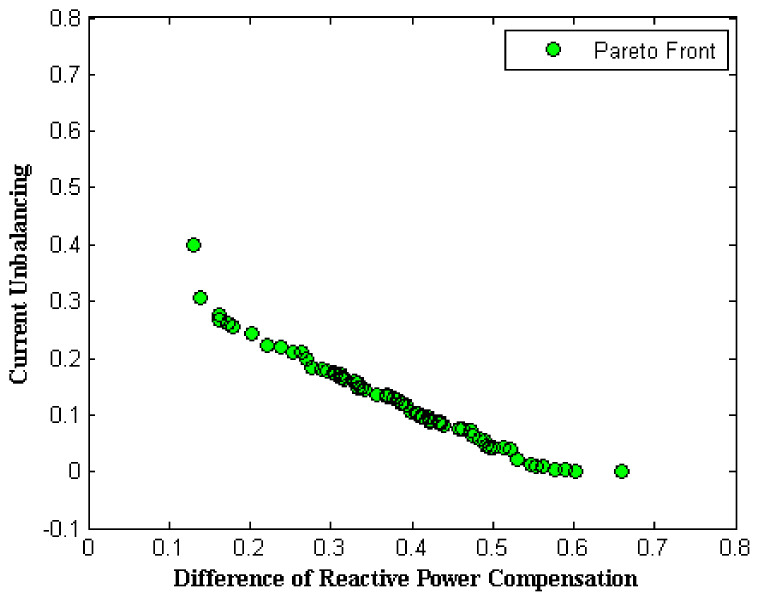
Determined Pareto Front.

Due to real-time operation and for better convergence of the optimization algorithm, Equations (15)–(19) were used for generating the initial population in the decision space. As mentioned, two separated objectives were considered that should be minimized. For this purpose, NSGA-II was utilized to determine the Pareto front. Figure 8 shows the optimal solutions in the objective space for a specific moment (for example, the tenth sec). In the figure, the optimal solutions of the objective functions are identified. Users can change *λ*_1_ and *λ*_2_ coefficients to change the preference between the two objectives based on the circumstances. Table 2 presents some feasible solutions derived from Pareto front individuals in both objective and decision spaces.

**Figure 9:**
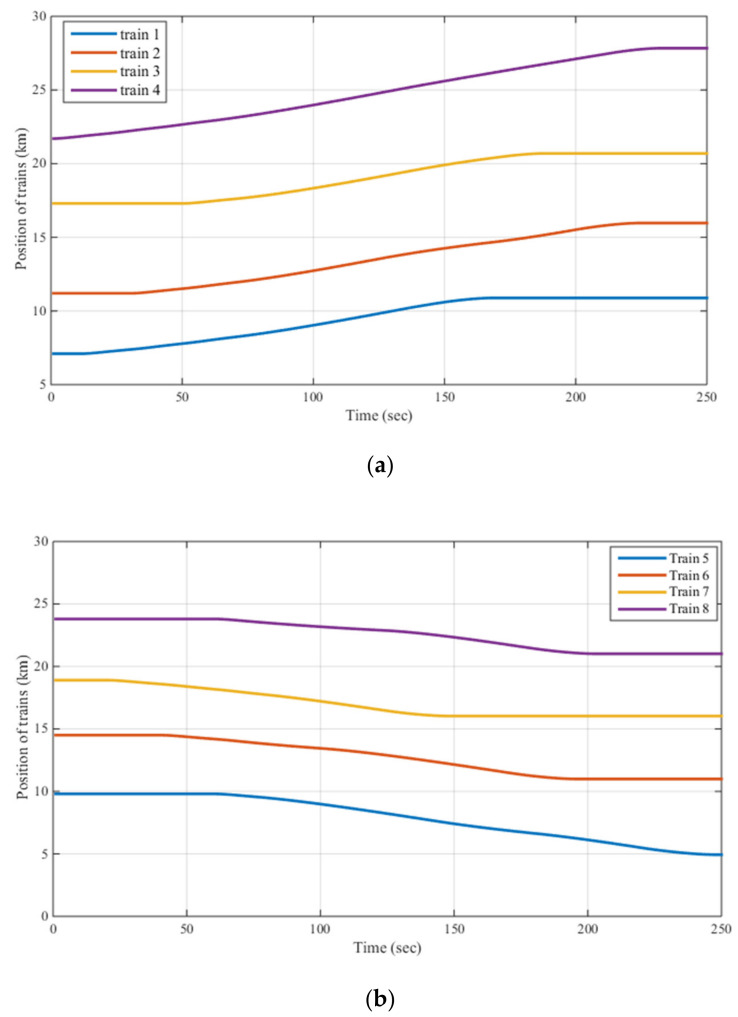

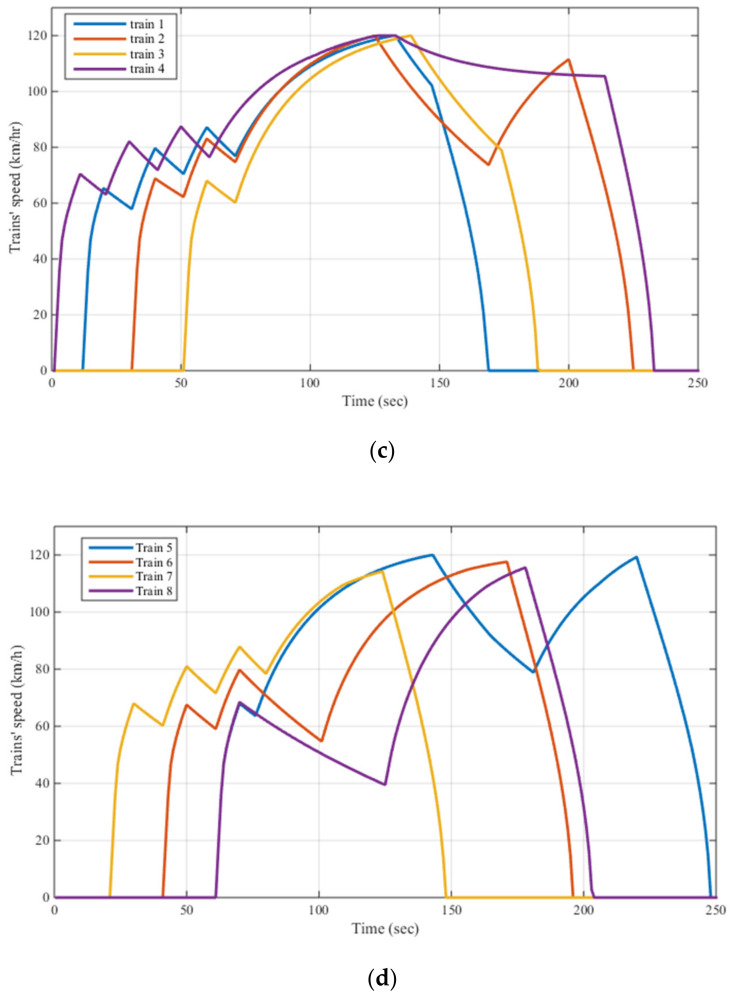
(**a**,**b**): timetable of trains moving on two opposite rail lines considered in the simulation; (**c**,**d**): speed profiles of the trains moving on two opposite rail lines.

It is necessary to note that sampling from the system was carried out every one second, and the optimization algorithm was always performed during a time shorter than one second. It can be seen from Table 2 that the total running time of the algorithm was 0.839 s, and this time satisfies the real-time requirement since the sampling time of the system is one second. This simulation was executed on a simulator of 2 × 25 kV autotransformer type electric traction system based on a timetable shown in Figure 9a,b. The simulation was performed with 250 sec time duration for various operational modes of some moving trains (Figure 9c,d) along a Multi-Transmission Line (MTL).

**Figure 10:**
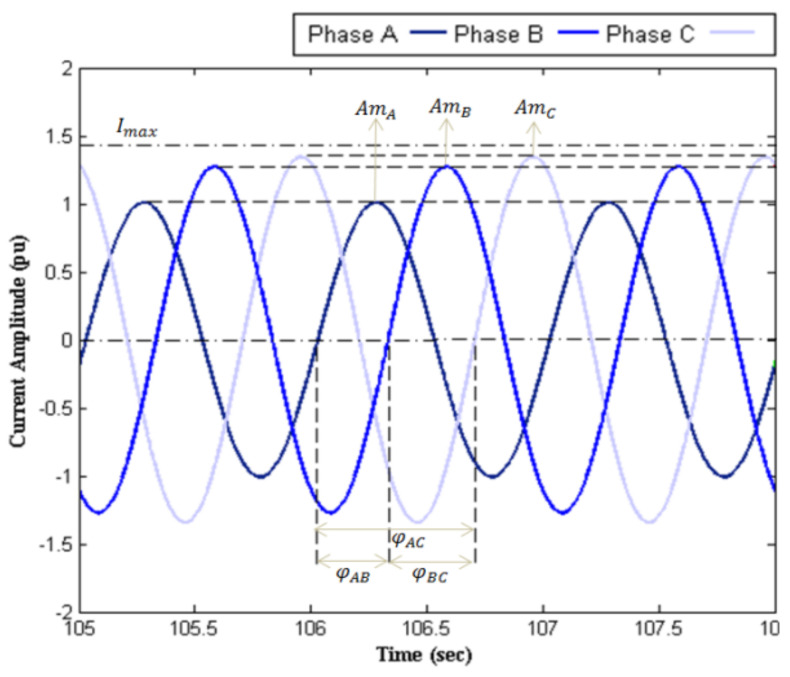
Network currents without SVC.

**Figure 11:**
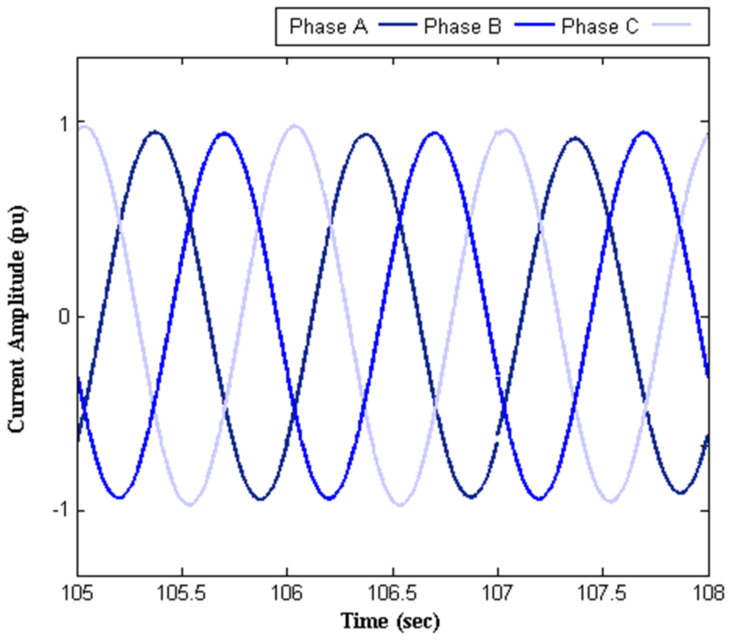
Network currents with SVC tunned by the proposed approach.

**Figure 12:**
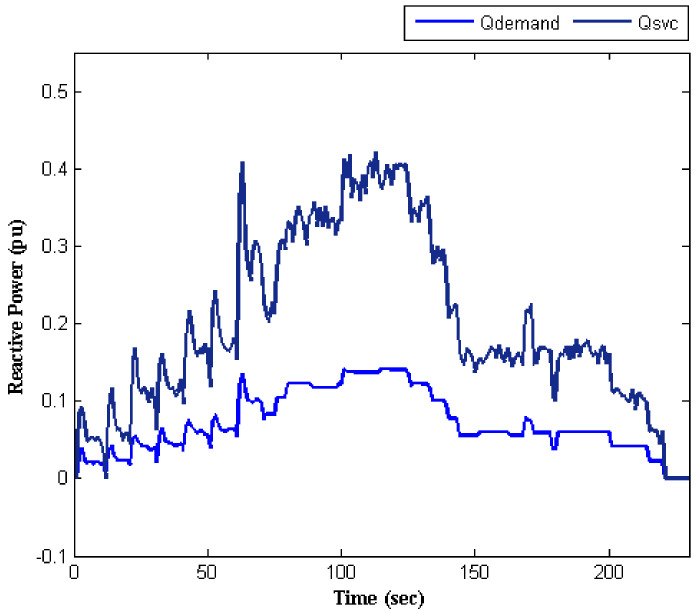
Reactive power profile.

**Figure 13:**
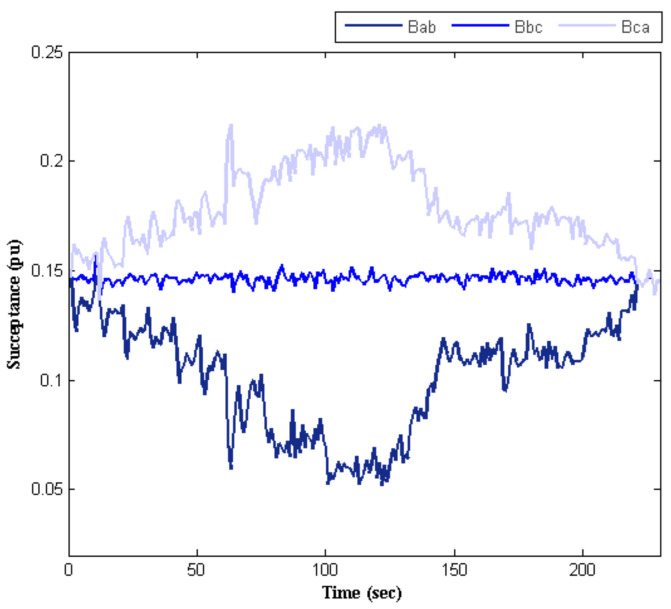
SVC’s succeptances profile based on the considered timetable.

Some results such as network currents (before and after SVC implementation), susceptance, and reactive power profiles are illustrated in Figure 10, Figure 11, Figure 12 and Figure 13, respectively. Figure 10 illustrates the currents of the network before applying the SVC. It is obvious that the electric traction system imposes a massive impact on the upstream network, which leads to a large unbalance. Figure 11 shows the SVC capability for reducing the mentioned problem. Table 3 shows the maximum deviations in amplitude and phase from desirable values before and after SVC installation. In this table, *Am_A_*, *Am_B_*, and *Am_C_* denote the amplitude of the three-phase currents, and *φ_AB_*, *φ_BC_*, and *φ_CA_* are the angle difference between the three-phase currents. Considerable mitigation of the unbalance in three-phase currents is observed due to the installation of SVC with proper parameters tuning. This leads to improvement in the power factor of the load as well as a reduction in the losses of the traction system. For instance, considering phase *A*, the percent of amplitude deviation is decreased from 23% to 1.8%. Moreover, a reduction in angle deviation from 8.5% to 1% is observed between phases *A* and *B* as a result of SVC installation. It should be noted that such deviations in amplitude and angle are very undesirable in high-load conditions.

As mentioned above, two independent objectives were considered. The first objective was mitigating the current unbalance that has been explained. The next objective was to provide reactive power to the electric traction system appropriately. Figure 12 shows that the SVC reactive power tracks the reactive power demanded by the traction system, as well. As shown in Figure 13, the values of the SVC succeptances were updated at every moment, which implies the system’s capability in adaptation to different conditions. This also indicates the flexibility of the proposed technique for tunning the SVC.

## 6. Conclusions

Due to imposing large current unbalance and consumption of variable reactive power, the traction system has a considerable impact on the whole network. In this paper, an SVC was considered in the traction substation to mitigate these problems. To achieve a favorable performance, it was proposed to determine the SVC optimum parameters using an intelligent optimization algorithm entitled NSGA-II. In this regard, a multi-objective optimization problem was defined mathematically to minimize network current unbalance and the difference between the demanded reactive power and the SVC reactive power. Two weighting factors were considered so that one can set the priority of the two objectives. Using the output solutions of NSGA-II, appropriate tunning of SVC parameters was realized. To evaluate the performance of the proposed approach, a comprehensive simulator for 2 × 25 kV AC traction systems was developed based on a mathematical model of different parts of the AC traction system. As the simulation scenario, a complete travel which means starting from a point and stopping at a destination by considering all movement modes (motoring, coasting, and braking), was allocated to all trains. The simulation results confirmed that even under the worst condition where all trains are in the accelerating mode, reduction in current unbalances and reactive power compensation can be realized effectively using the SVC tunned by the proposed approach. In other words, succeptances of the SVC are updated at every moment to adapt to the corresponding operating condition. Specifically, adjustment of SVC parameters can be performed such that the traction system is considered as a relatively constant load to the system. Moreover, it was shown that the performance of the proposed algorithm is real-time since the total running time of the algorithm is less than the sampling time. Due to the high running speed of NSGA-II in comparison with other multi-objective optimization techniques and the diversity of the solutions along the Pareto Front obtained by NSGA-II, optimum tunning of SVC can be achieved efficiently in a real-time manner to satisfy current unbalance reduction and reactive power compensation in a realistic traction system.

## Figures and Tables

**Table 1:** Specifications of the traction substation, autotransformer, and the trains in the simulation.

TSS		AT		Train	
Primary voltage	230 kV	Rated Voltage	25 kV	Rated Power	4 MVA
Secondary voltage	25 kV	Leakage Impedances	0.1564 + j0.0997 Ω	Power Factor	0.7
Rated Power	16.5 MVA	Rated Power	5 MVA	Mass	200 tons

**Table 2:** Some solutions derived from the determined Pareto front.

**Sol.**	**Objective Space**		**Decision Space**			**Running Time**
	**Obj. I**	**Obj. II**	$B_{AB}$	$B_{BC}$	$B_{CA}$	0.893 sec (in time interval (910) sec of simulator running)
1	0.050135	0.309336	0.147019	0.194459	0.228315	
2	0.245369	0.182319	0.182085	0.197768	0.214491	
3	0.127370	0.258360	0.160946	0.196607	0.222621	
4	0.252364	0.177715	0.185536	0.197619	0.216171	
5	0.054233	0.306107	0.149850	0.196143	0.229902	
6	0.312023	0.139873	0.201127	0.197404	0.217196	
7	0.320461	0.134344	0.199656	0.198789	0.213597	
8	0.465730	0.039019	0.223266	0.199745	0.200516	
9	0.339299	0.121827	0.194002	0.199469	0.203125	
10	0.271779	0.165950	0.193625	0.197261	0.219731	

**Table 3:** Maximum deviations in amplitude and phase of currents.

Without SVC	Amplitude Deviation	$\Delta Am_{A}$	−23%
		$\Delta Am_{B}$	−9.9%
		$\Delta Am_{C}$	−4.8%
	Phase Deviation	$\Delta \varphi_{AB}$	−8.5%
		$\Delta \varphi_{BC}$	+11.6%
		$\Delta \varphi_{CA}$	+ 4.1%
With SVC	Amplitude Deviation	$\Delta Am_{A}$	−1.8%
		$\Delta Am_{B}$	−0.2%
		$\Delta Am_{C}$	+0.9%
	Phase Deviation	$\Delta \varphi_{AB}$	+1%
		$\Delta \varphi_{BC}$	−0.5%
		$\Delta \varphi_{CA}$	+1.5%

## Data Availability

The data present in this study are available on request from the author M.H.B.

## Appendix A

The following figure presents a view of the developed simulator. This simulator is sectionalized into several parts. The upper part is allocated for training settings. Electrical parameters curves can be shown dynamically in the monitoring section. As some load-flow methods are developed, the method for performing the load-flow analysis can be selected in the load flow section. The measurement part shows every electric parameter of the system. In the optimization section, objective functions and other settings are selected, and ultimately, the optimization process and results are displayed dynamically, which provides a fine scheme for analyzing the results of optimization while the simulation of the overall traction system is carried on.
